# Supplementary material for: Type 1 interferons and Foxo1 down-regulation play a key role in age-related T-cell exhaustion in mice
Source: Nat Commun. 2024 Feb 26;15:1718. doi: 10.1038/s41467-024-45984-8 (PMC10897180; doi:10.1038/s41467-024-45984-8)
Supplement: Supplementary file 1 — Supplementary Information [file 41467_2024_45984_MOESM1_ESM.pdf]

Supplementary Information related to:

Type 1 interferons and Foxo1 down-regulation play a key role in age-related T-cell exhaustion in mice

By

Aurélie Durand, Nelly Bonilla, Théo Level, Zoé Ginestet, Amélie Lombès, Vincent Guichard, Mathieu Germain, Sébastien Jacques, Franck Letourneur, Marcio Do Cruzeiro, Carmen Marchiol, Gilles Renault, Morgane Le Gall, Céline Charvet, Agnès Le Bon, Bruno Martin, Cédric Auffray and Bruno Lucas

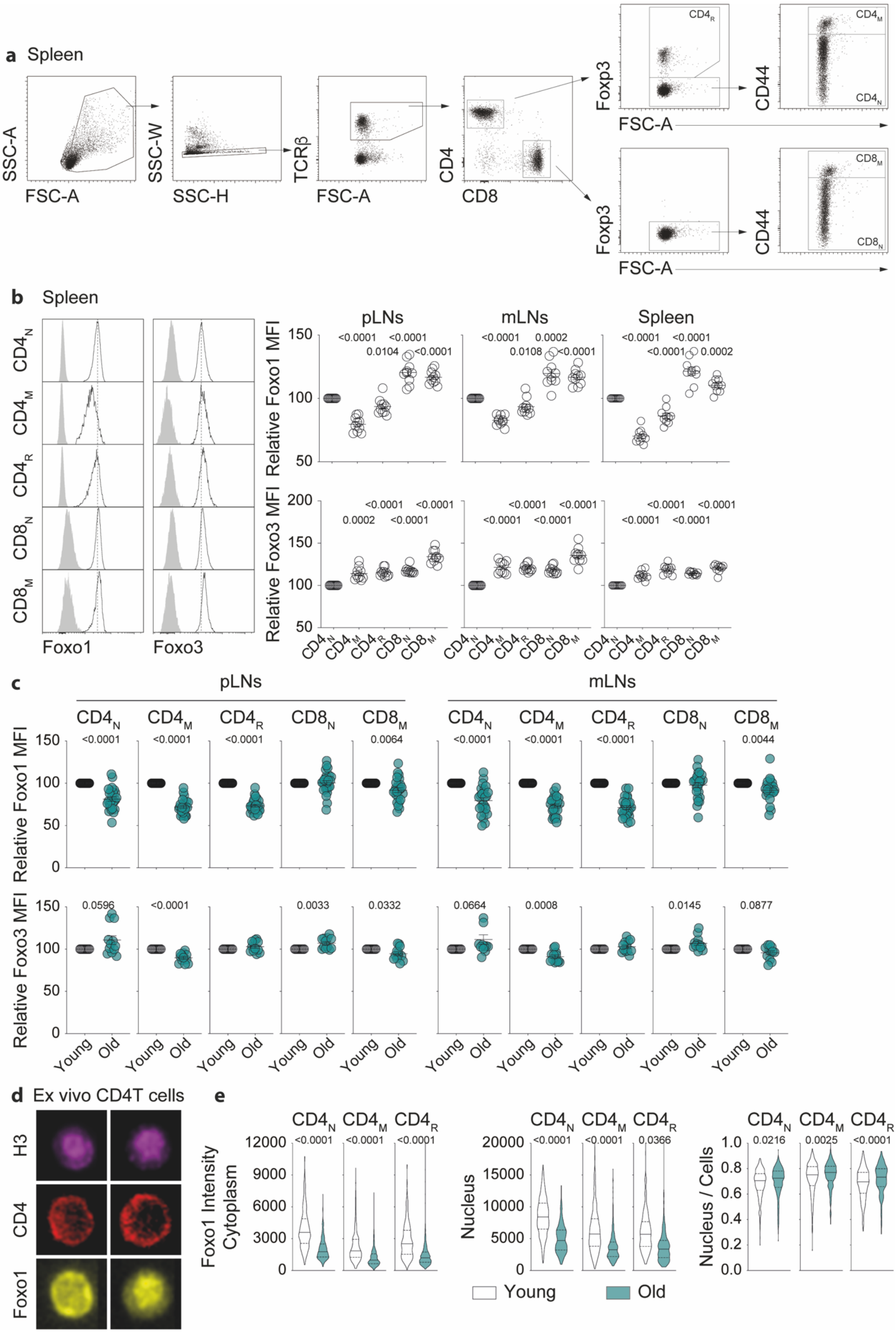
a Spleen
SSC-A
FSC-A
SSC-W
SSC-H
TCRβ
FSC-A
CD4
CD8
Foxp3
FSC-A
CD44
b Spleen
Foxo1
Foxo3
pLNs
mLNs
Spleen
Relative Foxo1 MFI
Relative Foxo3 MFI
c
pLNs
mLNs
Relative Foxo1 MFI
Relative Foxo3 MFI
Young
Old
d Ex vivo CD4T cells
H3
CD4
Foxo1
e
Foxo1 Intensity Cytoplasm
Nucleus
Nucleus / Cells
Young
Old

Figure S1

Fig. S1 Gradual down-regulation of Foxo1 in T cells with age. a, Gating strategy to define regulatory CD4 T cells ($CD4_R$), memory CD4 T cells ($CD4_M$), naive CD4 T cells ($CD4_N$), memory CD8 T cells ($CD8_M$) and naive CD8 T cells ($CD8_N$). **b,** Foxo1 and Foxo3 fluorescence histograms of the indicated T-cell subsets from the spleen of a representative young adult mouse are shown (left panel). Grey histograms correspond to Florescence Minus One (FMO) controls. Relative MFIs were calculated by dividing the MFI of a given T-cell subset by the MFI of $CD4_N$ cells (right panel) and are shown for the spleen, peripheral (pLNs) and mesenteric (mLNs) lymph-nodes of young adult mice. **c,** Foxo1 and Foxo3 relative MFIs are shown for the indicated T-cell subsets from pLNs and mLNs as a function of mouse age. Relative MFIs were calculated by dividing the MFI of a given T-cell subset of the old mouse by the MFI of the same T-cell subset of the barcoded young adult mouse. **d,e,** Lymph-node cells from young adult and aged mice were analyzed using the Imagestream flow imager. Representative staining (**d**) and quantification of nuclear and cytoplasmic Foxo1 (**e**) are shown. Quantifications are represented as Means ± SEM. The significance of differences between two series of results was assessed using Student's paired (**b**) or unpaired (**c** and **e**) t test. Significant ($p < 0.05$) or almost significant ($0.05 < p < 0.10$) p-values are indicated. Source data are provided as a Source Data file.

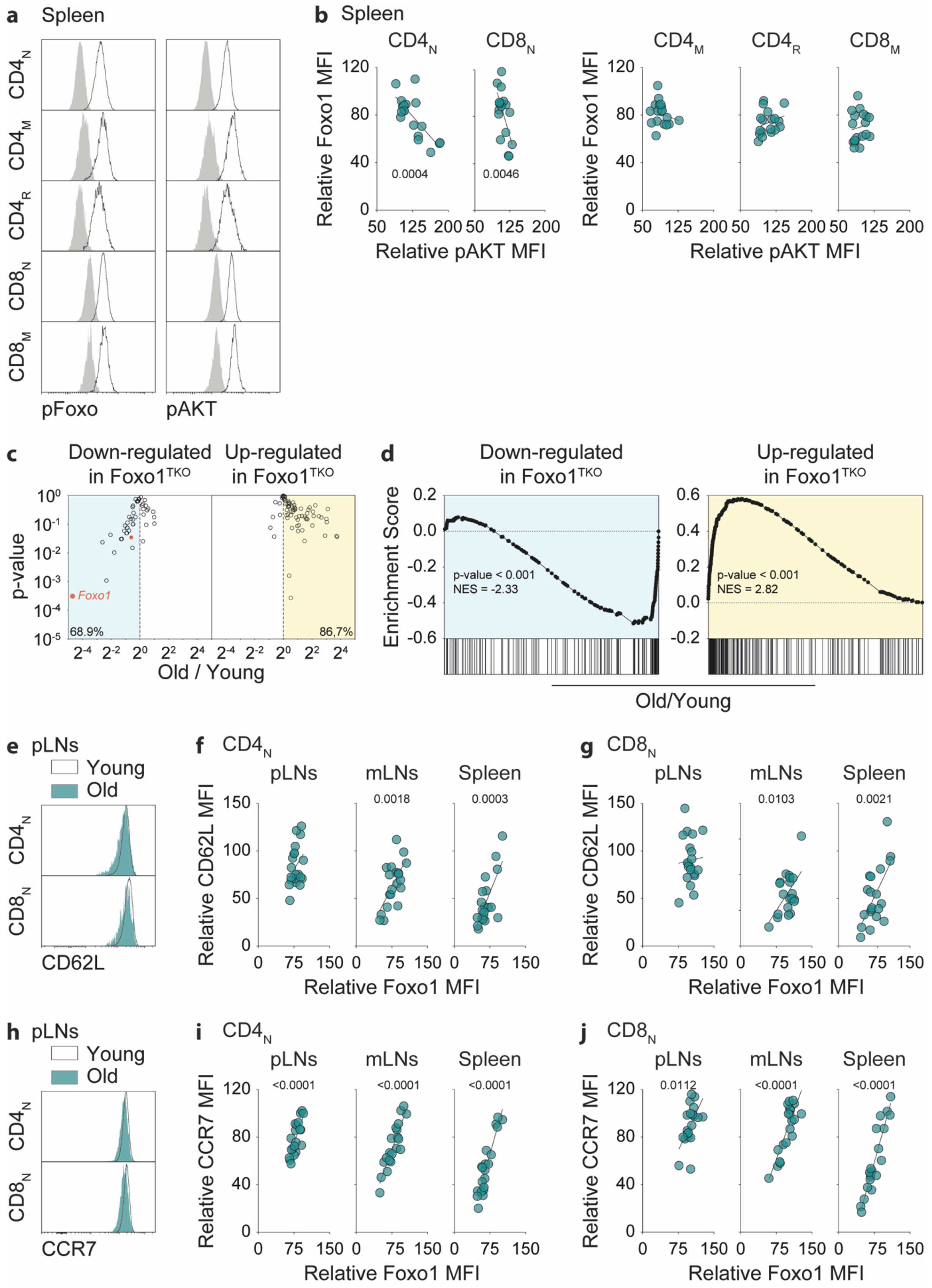
a Spleen
CD4N
CD4M
CD4R
CD8N
CD8M
pFoxo
pAKT
b Spleen
CD4N CD8N CD4M CD4R CD8M
Relative Foxo1 MFI
0.0004
0.0046
Relative pAKT MFI
c
Down-regulated in Foxo1TKO
Up-regulated in Foxo1TKO
p-value
Foxo1
68.9%
86,7%
Old / Young
d
Down-regulated in Foxo1TKO
Up-regulated in Foxo1TKO
Enrichment Score
p-value < 0.001
NES = -2.33
p-value < 0.001
NES = 2.82
Old/Young
e pLNs
Young
Old
CD62L
f CD4N
g CD8N
pLNs mLNs Spleen
0.0018
0.0003
0.0103
0.0021
Relative CD62L MFI
Relative Foxo1 MFI
h pLNs
Young
Old
CCR7
i CD4N
j CD8N
<0.0001
0.0112
Relative CCR7 MFI
Relative Foxo1 MFI

Figure S2

Fig. S2 Foxo1 down-regulation in T cells with age imprints their transcriptionnal signature. a, pFoxo and pAKT fluorescence histograms of the indicated T-cell subsets from the spleen of a representative young adult mouse are shown (left panel). Grey histograms correspond to Florescence Minus One (FMO) controls. **b,** Relative expression of Foxo1 by the indicated T-cell subsets from the spleen of old mice as a function of pAKT relative expression. **c,** Same as in Fig. 1g with p value of < 0.05 and fold change > 2 or < -2 to determine the list of genes down- or up-regulated in $CD4_N$ cells from $Foxo1^{TKO}$ mice. **d,** GSEA analysis visualizing the expression of genes down- or up-regulated in $CD4_N$ cells from $Foxo1^{TKO}$ mice as a function of mouse age. **e-j,** Relative expression of CCR7 and CD62L by $CD4_N$ and $CD8_N$ cells from the SLOs of old mice. **e, h,** CD62L (**e**) and CCR7 (**h**) fluorescence histograms of $CD4_N$ and $CD8_N$ cells from the pLNs of a representative young adult versus old mouse are shown. **f, g,** Relative expression of CD62L by $CD4_N$ (**f**) and $CD8_N$ (**g**) cells from the SLOs of old mice as a function of Foxo1 relative expression. **i, j,** Relative expression of CCR7 by $CD4_N$ (**i**) and $CD8_N$ (**j**) cells from the SLOs of old mice as a function of Foxo1 relative expression. Relative MFIs were calculated by dividing the MFI of a given T-cell subset of the old mouse by the MFI of the same T-cell subset of the barcoded young adult mouse. For assessing correlations, Pearson correlation (two-sided test, coefficient and 95% confidence intervals) was used (**b**, **f**, **g**, **i** and **j**). The p-values for the GSEA test statistics are calculated by permutation (**d**). Significant ($p < 0.05$) p-values are indicated. Source data are provided as a Source Data file.

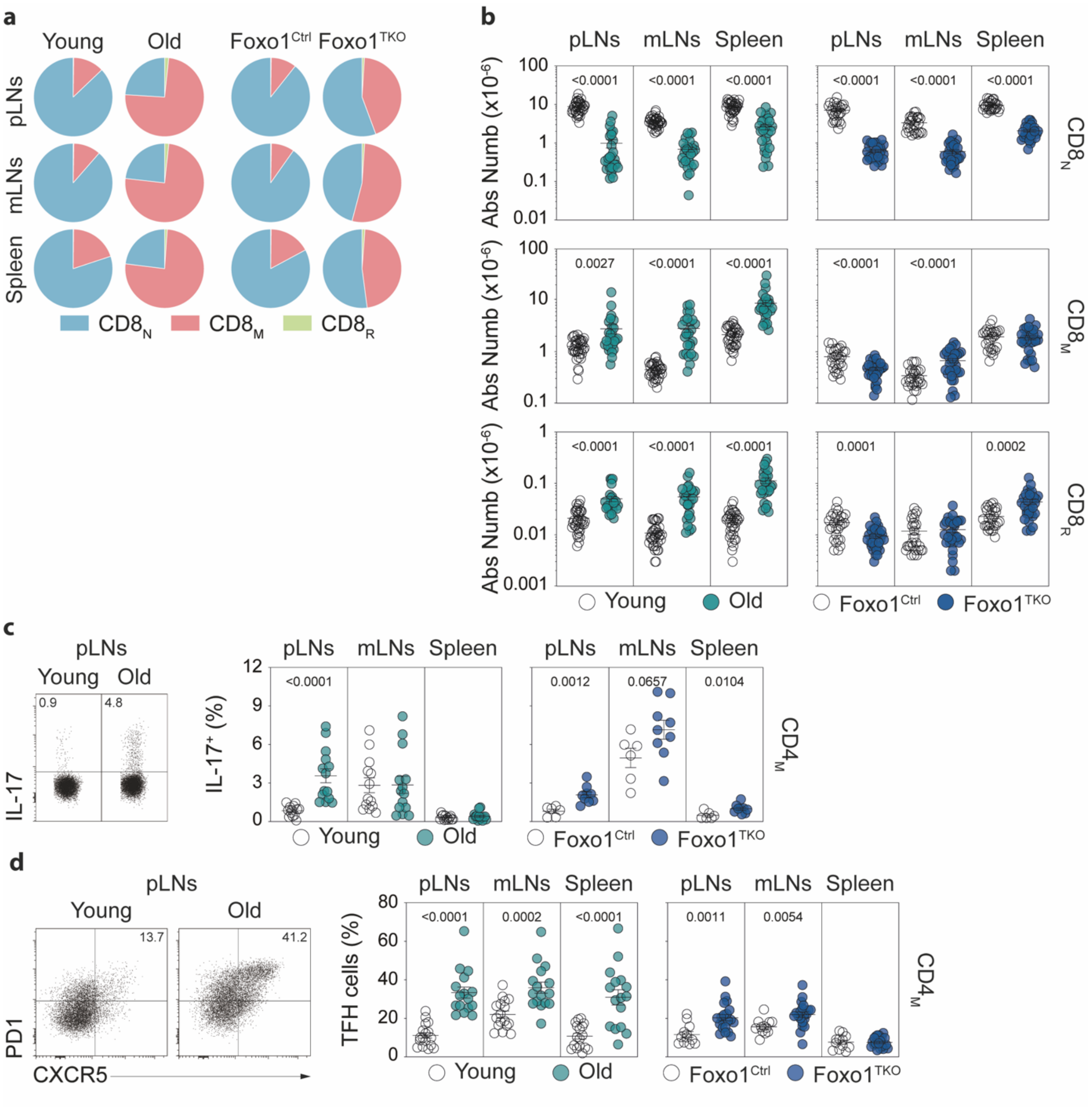

Figure S3

Fig. S3 $CD4_M$ and $CD8_M$ cells of young adult Foxo1^{TKO} mice and old wild-type mice share many features. a, Distribution of $CD8_N$, $CD8_M$ and $CD8_R$ cells among CD8 T cells in the indicated SLOs of young adult and old WT mice versus young adult Foxo1^{Ctrl} and Foxo1^{TKO} mice. **b,** Total cell numbers of $CD8_N$, $CD8_M$ and $CD8_R$ cells recovered from the SLOs of the indicated mice. **c,** Representative IL-17 staining is shown for pLN CD4 T cells from the indicated representative mice (left panel). Percentages of IL-17-producing cells among $CD4_M$ cells are shown for the spleen, peripheral (pLNs) and mesenteric (mLNs) lymph-nodes of the indicated mice (right panel). **d,** PD1/CXCR5 representative dot-plots are shown for pLN CD4 T cells from the indicated representative mice (left panel). Percentages of TFH cells (PD1$^+$ CXCR5$^+$) among $CD4_M$ cells are shown for the spleen, peripheral (pLNs) and mesenteric (mLNs) lymph-nodes of the indicated mice (right panel). Quantifications are represented as Means ± SEM. The significance of differences between two series of results was assessed using Student's unpaired t test (**b-d**). Significant ($p < 0.05$) or almost significant ($0.05 < p < 0.10$) p-values are indicated. Source Data are provided as a Source Data file.

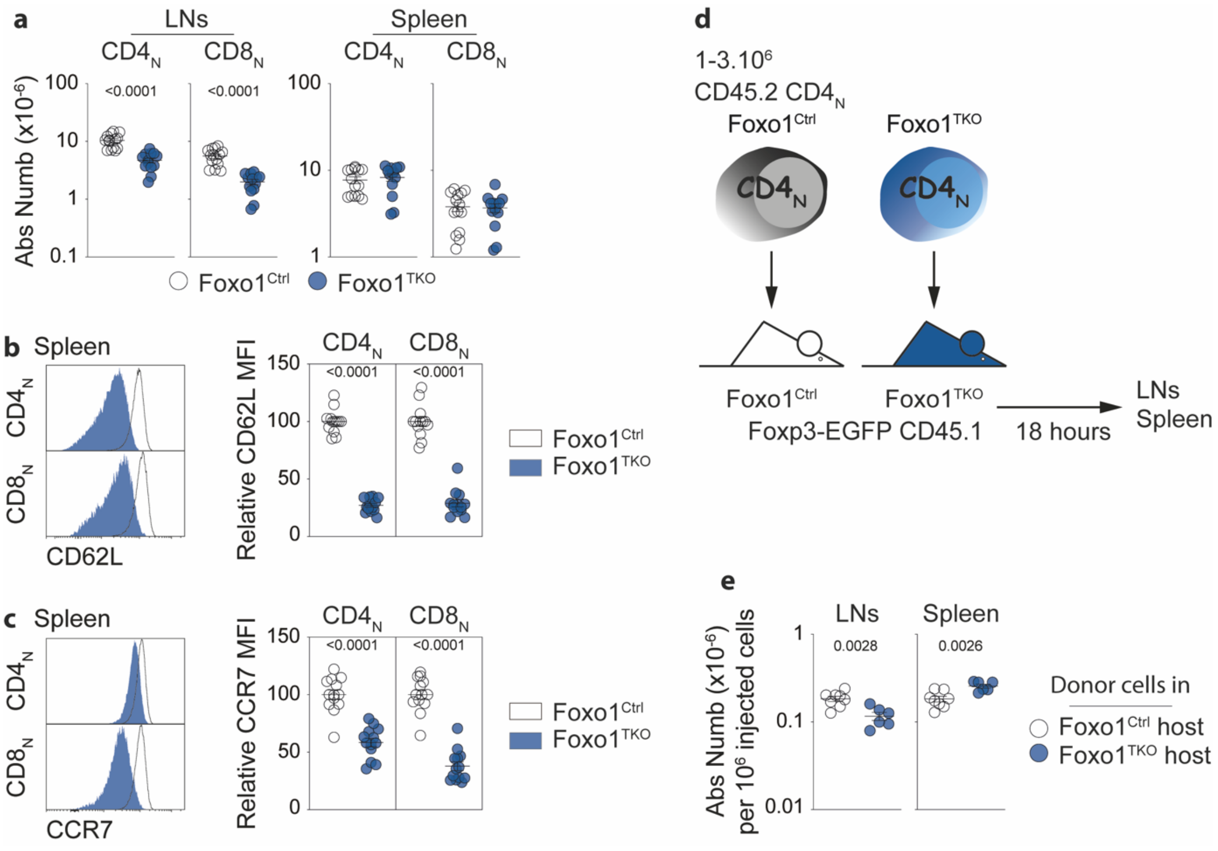

Figure S4

Fig. S4 Altered homing of Foxo1-deficient naïve T cells into lymph-nodes a, Absolute cell numbers of $CD4_N$ and $CD8_N$ cells from the spleen and lymph-nodes of 1-month-old $Foxo1^{Ctrl}$ and $Foxo1^{TKO}$ mice. **b, c,** CD62L (**b**) and CCR7 (**c**) fluorescence histograms of $CD4_N$ and $CD8_N$ cells from the spleen of a representative $Foxo1^{Ctrl}$ mouse and a representative $Foxo1^{TKO}$ mouse are shown (left panels). Quantification of the relative expression of CD62L (**b**) and CCR7 (**c**) by $CD4_N$ and $CD8_N$ cells from the spleen. Relative expression of CD62L or CCR7 was calculated by dividing the MFI of a given T-cell subset by the average MFI obtained from the same T-cell subset in $Foxo1^{Ctrl}$ mice. **d, e,** 1-3.10^6 $CD4_N$ cells from the LNs of 1-month-old CD45.2 $Foxo1^{TKO}$ or $Foxo1^{Ctrl}$ mice were injected i.v. into 1-month-old CD45.1 $Foxo1^{TKO}$ or $Foxo1^{Ctrl}$ mice respectively. Recipient mice were sacrificed 18 hours later. Diagram illustrating the experimental model (**d**). Absolute cell numbers of CD4 donor T cells recovered from the indicated SLOs of recipient mice per 10^6 injected cells (**e**). Quantifications are represented as Means ± SEM. The significance of differences between two series of results was assessed using Student's unpaired t test (**a-c** and **e**). Significant ($p < 0.05$) p-values are indicated. Source data are provided as a Source Data file.

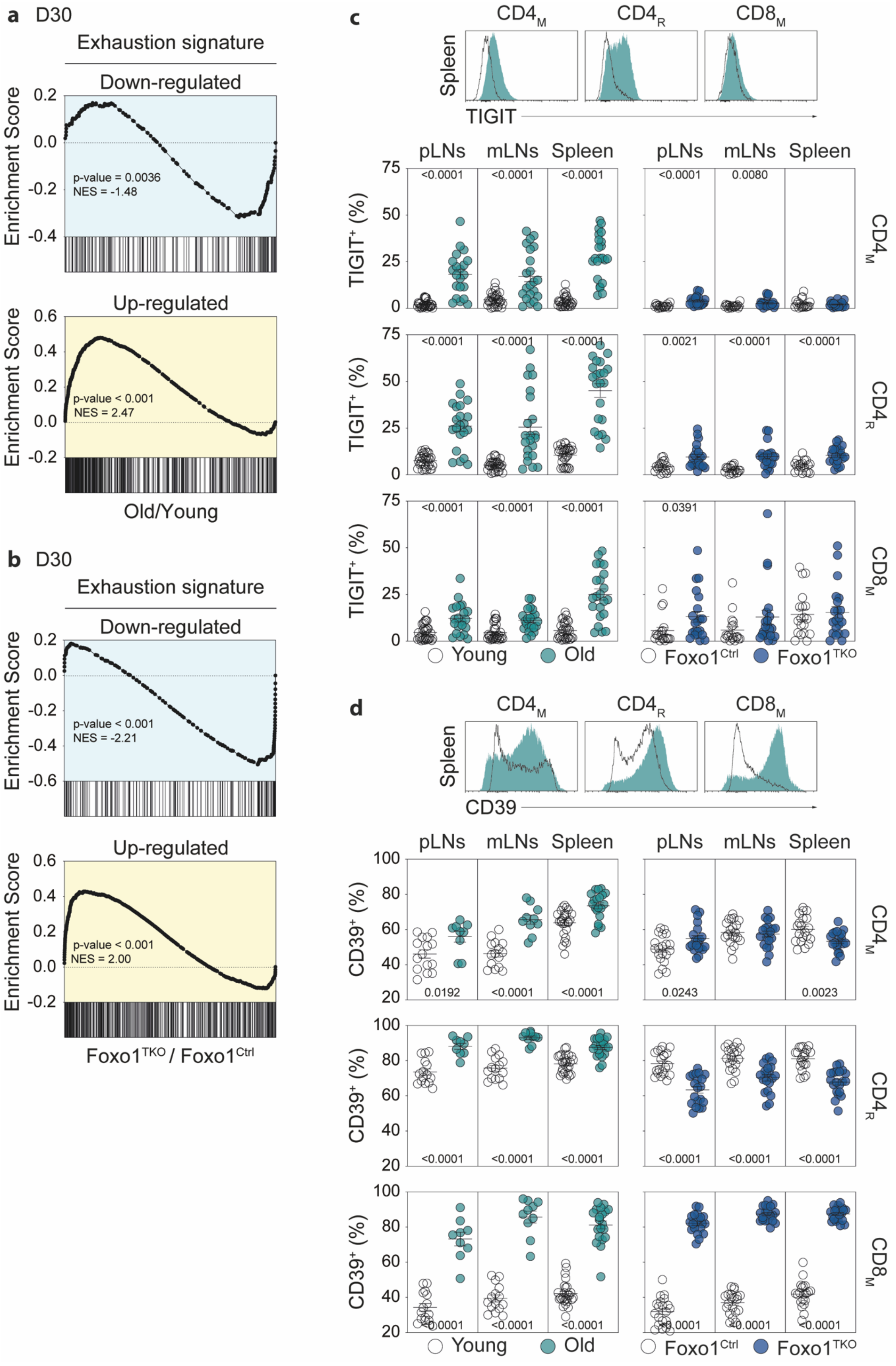
a D30
Exhaustion signature
Down-regulated
Enrichment Score
p-value = 0.0036
NES = -1.48
Up-regulated
p-value < 0.001
NES = 2.47
Old/Young
b D30
Exhaustion signature
Down-regulated
p-value < 0.001
NES = -2.21
Up-regulated
p-value < 0.001
NES = 2.00
Foxo1TKO / Foxo1Ctrl
c
CD4M CD4R CD8M
Spleen
TIGIT
pLNs mLNs Spleen pLNs mLNs Spleen
TIGIT+ (%)
Young Old Foxo1Ctrl Foxo1TKO
d
CD39
CD39+ (%)

Figure S5

Fig. S5 Memory and Regulatory T cells from the SLOs of old WT and young adult Foxo1^{TKO} mice are exhausted. a, GSEA analysis visualizing the expression of genes down- or up-regulated in exhausted Day 30 T cells (GSE30431) by CD4$_N$ cells from old versus young adult mice. **b,** Same as in **a** for CD4$_N$ cells from Foxo1^{TKO}/Foxo1^{Ctrl} mice. **c**, TIGIT fluorescence histograms of the indicated T-cell subsets from the spleen of a representative old mouse and a representative young adult mouse are shown (upper panel). Percentages of TIGIT$^+$ cells among CD4$_M$, CD4$_R$ and CD8$_M$ cells are shown for the indicated SLOs of old/young adult mice (lower left panel) and Foxo1^{TKO}/Foxo1^{Ctrl} mice (lower right panel). **d,** Same as in **c** for CD39. Quantifications are represented as Means ± SEM. The p-values for the GSEA test statistics are calculated by permutation (**a**, **b**). The significance of differences between two series of results was assessed using Student's unpaired t test (**c** and **d**). Significant ($p < 0.05$) p-values are indicated. Source data are provided as a Source Data file.

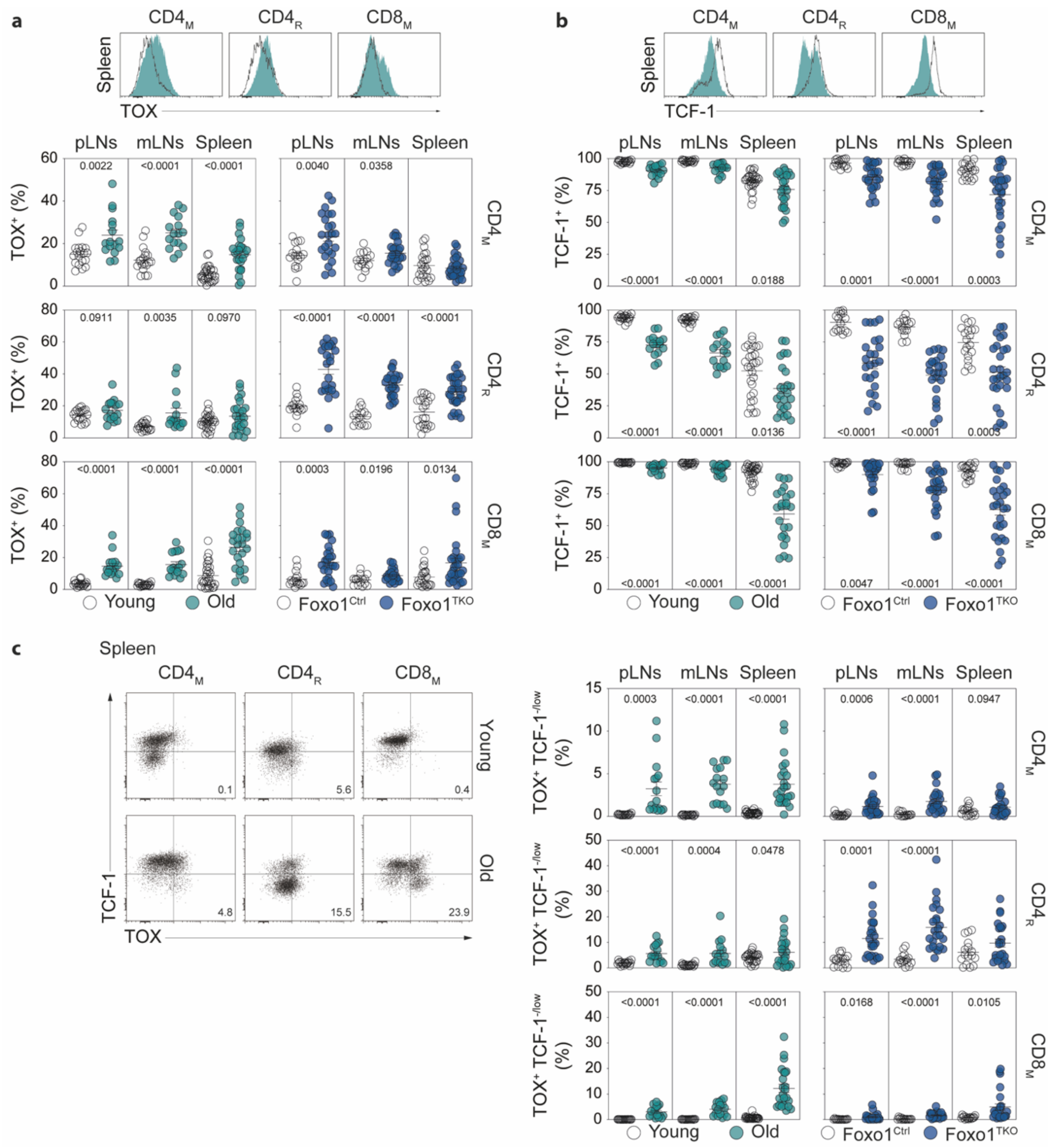

Figure S6

Fig. S6 Memory and Regulatory T cells from the SLOs of old WT and young adult Foxo1^{TKO} mice contain a significant proportion of TOX$^+$ TCF-1$^{-/low}$ terminally differentiated exhausted cells. a, TOX fluorescence histograms of the indicated T-cell subsets from the spleen of a representative old mouse and a representative young adult mouse are shown (upper panel). Percentages of TOX$^+$ cells among CD4$_M$, CD4$_R$ and CD8$_M$ cells are shown for the indicated SLOs of old/young adult mice (lower left panel) and Foxo1^{TKO}/Foxo1^{Ctrl} mice (lower right panel). **b,** Same as in **a** for TCF-1. **c,** TOX/TCF-1 representative dot-plots are shown for spleen CD4 T cells from the indicated representative mice (left panel). Percentages of TOX$^+$ TCF-1$^{-/low}$ cells among CD4$_M$, CD4$_R$ and CD8$_M$ cells are shown for the indicated SLOs of old/young adult mice (middle panel) and Foxo1^{TKO}/Foxo1^{Ctrl} mice (right panel). Quantifications are represented as Means ± SEM. The significance of differences between two series of results was assessed using Student's unpaired t test (**a-c**). Significant ($p < 0.05$) or almost significant ($0.05 < p < 0.10$) p-values are indicated. Source data are provided as a Source Data file.

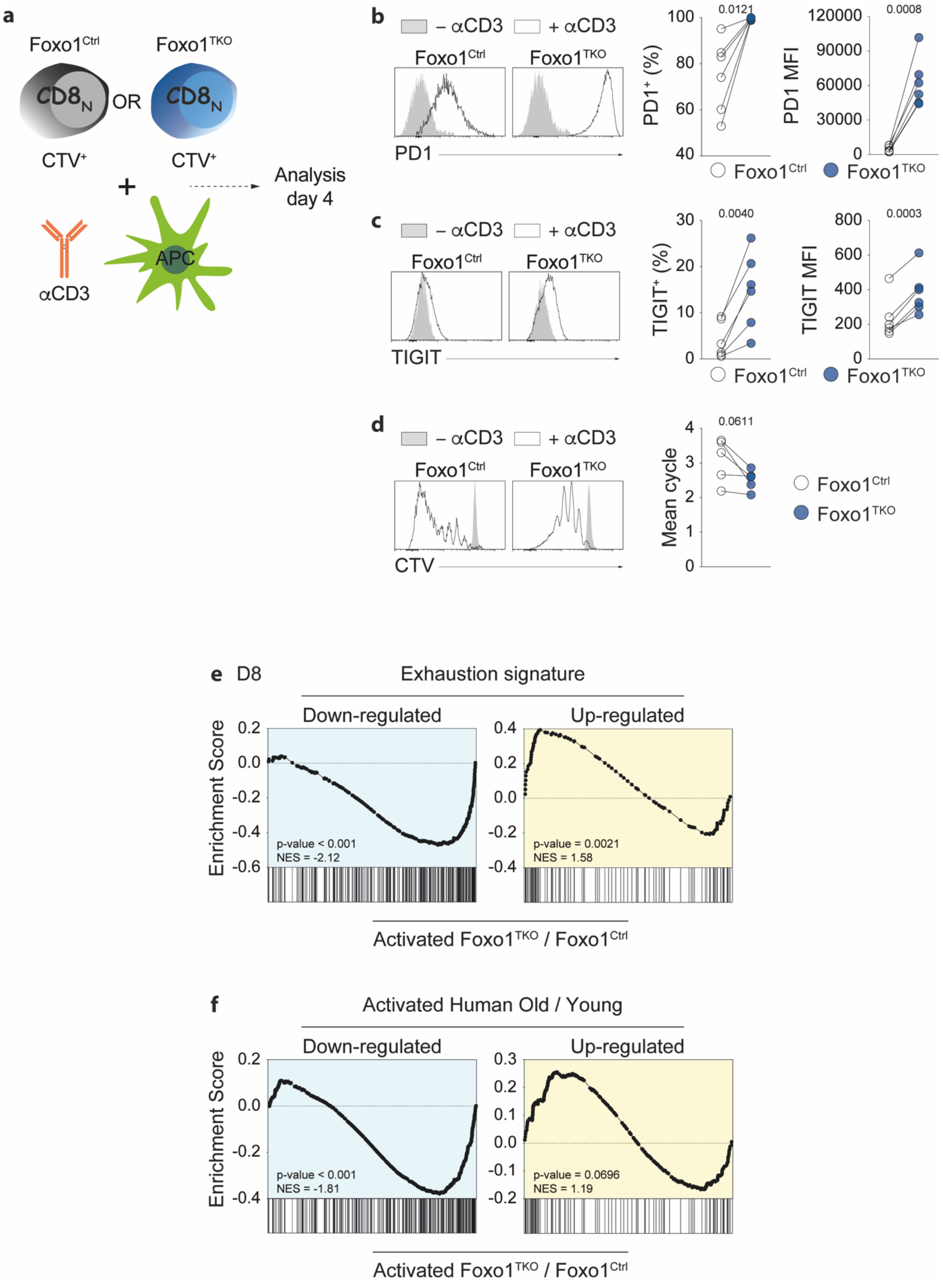
a
Foxo1Ctrl
Foxo1TKO
CD8N
OR
CD8N
CTV+
CTV+
Analysis day 4
αCD3
APC
b
− αCD3
+ αCD3
Foxo1Ctrl
Foxo1TKO
PD1
PD1+ (%)
PD1 MFI
0.0121
0.0008
c
TIGIT
TIGIT+ (%)
TIGIT MFI
0.0040
0.0003
d
CTV
Mean cycle
0.0611
e D8
Exhaustion signature
Down-regulated
Up-regulated
Enrichment Score
p-value < 0.001
NES = -2.12
p-value = 0.0021
NES = 1.58
Activated Foxo1TKO / Foxo1Ctrl
f
Activated Human Old / Young
Down-regulated
Up-regulated
Enrichment Score
p-value < 0.001
NES = -1.81
p-value = 0.0696
NES = 1.19
Activated Foxo1TKO / Foxo1Ctrl

Figure S7

Fig. S7 A link between Foxo1 deficiency and T-cell exhaustion. a-d, CTV-labeled purified $CD8_N$ cells from $Foxo1^{TKO}$ and $Foxo1^{Ctrl}$ mice were stimulated for 4 days with anti-CD3 in the presence of splenocytes from $CD3_{\varepsilon}KO$ mice. Diagram illustrating the experimental model (**a**). PD1 (**b**) and TIGIT (**c**) fluorescence histograms of T cells after 4 days of culture are shown for a representative experiment (left panel). Percentages of $PD1^+$ cells and PD1 MFI (**b**, n=6 independent experiments) and Percentages of $TIGIT^+$ cells and TIGIT MFI (**c,** n=6 independent experiments) among the progeny of $CD4_N$ cells after 4 days of culture (right panel). CTV fluorescence histograms of T cells after 4 days of culture are shown for a representative experiment (**d**, left panel). The average number of cell cycles was calculated and plotted (**d**, right panel, n=5 independent experiments). Each pair of dots represents an individual experiment. **e**, GSEA analysis visualizing the expression of genes down- or up-regulated in CD4 T cells 8 days after LCMV infection (GSE30431 Day 8) by the progeny of activated $CD4_N$ cells from $Foxo1^{TKO}$ versus $Foxo1^{Ctrl}$ mice after 4 days of culture. **f,** GSEA analysis visualizing the expression of genes down- or up-regulated in activated human T cells (SRP158502) as a function of age by the progeny of $CD4_N$ cells from $Foxo1^{TKO}$ versus $Foxo1^{Ctrl}$ mice after 4 days of culture. The significance of differences between two series of results was assessed using Student's paired t test (**b-d**). The p-values for the GSEA test statistics are calculated by permutation (**e**, **f**). Significant ($p < 0.05$) or almost significant ($0.05 < p < 0.10$) p-values are indicated. Source data are provided as a Source Data file.

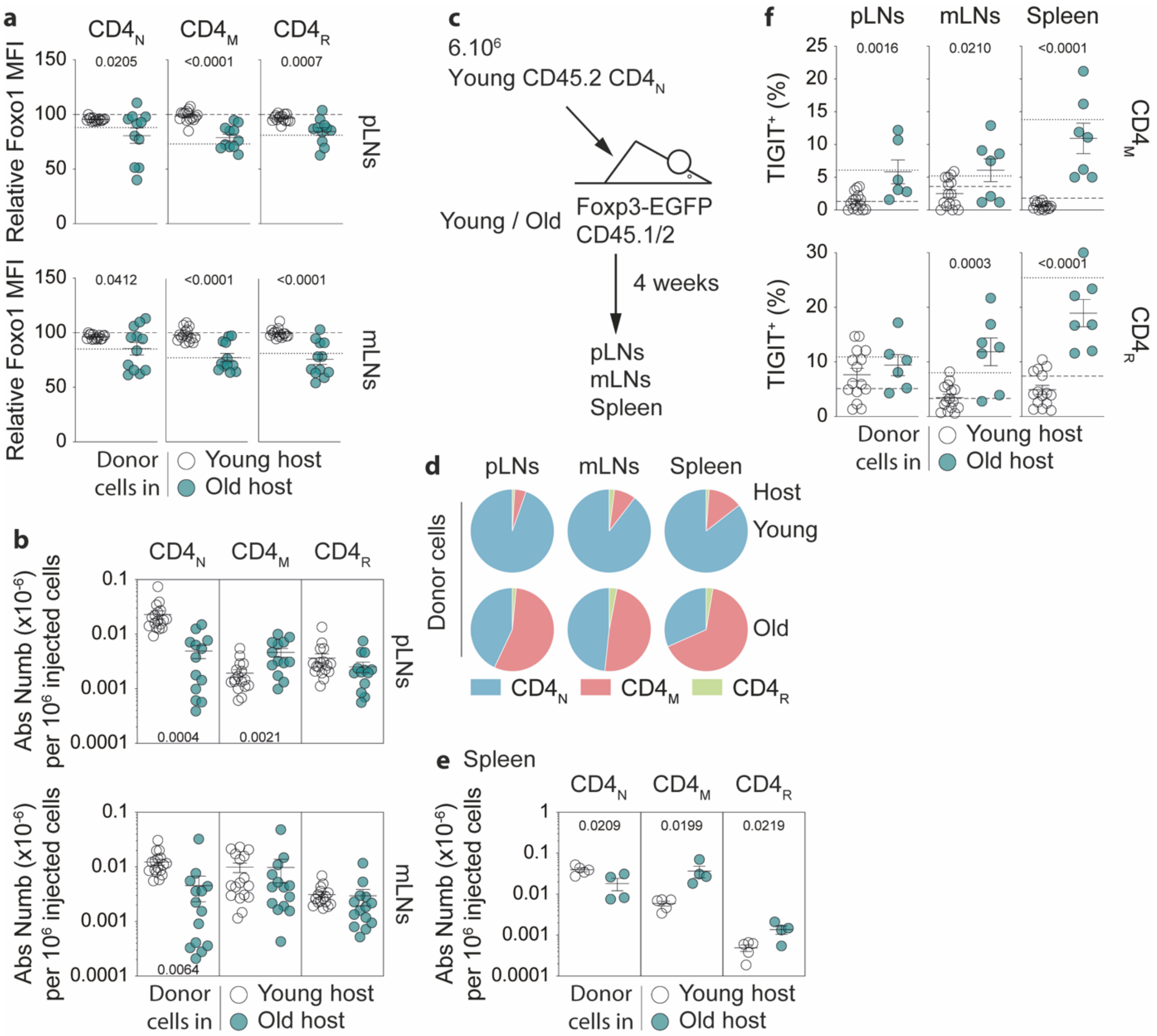

Figure S8

Fig. S8 T-cell-extrinsic signals participate to T-cell aging. a, Relative Foxo1 MFIs for $CD4_N$, $CD4_M$ and $CD4_R$ donor cells recovered from the pLNs and mLNs of recipient mice are shown. The dashed lines represent the average Foxo1 MFI of the corresponding T-cell subset from the pLNs and mLNs of young adult (----) and old (….) recipient mice. **b,** Absolute cell numbers of $CD4_N$, $CD4_M$ and $CD4_R$ donor cells recovered from pLNs and mLNs of young adult versus old recipient mice per 10^6 injected cells. **c,** Diagram illustrating the experimental model. **d,** Distribution of $CD4_N$, $CD4_M$ and $CD4_R$ cells among donor CD4 T cells recovered from the indicated SLOs of young adult versus old recipient mice. **e,** Absolute cell numbers of $CD4_N$, $CD4_M$ and $CD4_R$ donor cells recovered from the spleen of young adult versus old recipient mice per 10^6 injected cells. Panels **d** and **e** are related to the experimental model depicted in **c**. **f,** Percentages of $TIGIT^+$ cells among $CD4_M$ and $CD4_R$ donor cells are shown for the indicated SLOs of old versus young adult recipient mice. The dashed lines represent the average percentages for the corresponding T-cell subset of young adult (----) and old (….) recipient mice. Quantifications are represented as Means ± SEM. The significance of differences between two series of results was assessed using Student's unpaired t test (**a**, **b**, **e** and **f**). Significant ($p < 0.05$) p-values are indicated. Source Data are provided as a Source Data file.

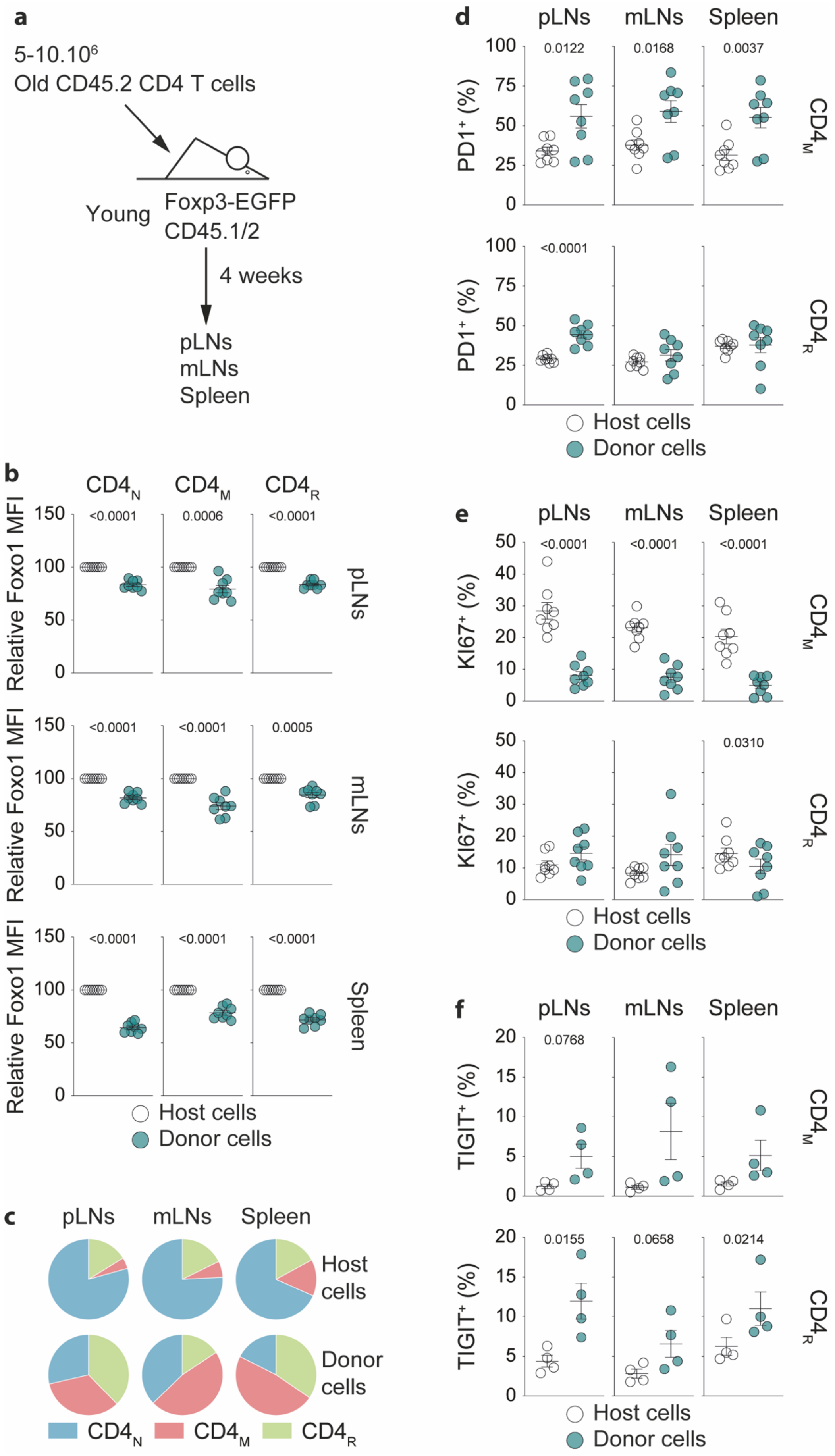
a
5-10.10^6
Old CD45.2 CD4 T cells
Young
Foxp3-EGFP
CD45.1/2
4 weeks
pLNs
mLNs
Spleen
b
CD4N
CD4M
CD4R
Relative Foxo1 MFI
pLNs
mLNs
Spleen
<0.0001
0.0006
0.0005
Host cells
Donor cells
c
pLNs
mLNs
Spleen
Host cells
Donor cells
CD4N
CD4M
CD4R
d
PD1+ (%)
0.0122
0.0168
0.0037
<0.0001
e
KI67+ (%)
0.0310
f
TIGIT+ (%)
0.0768
0.0155
0.0658
0.0214

Figure S9

Fig. S9 T-cell aging is not reversible. a, Diagram illustrating the experimental model. **b,** Relative Foxo1 MFIs for $CD4_N$, $CD4_M$ and $CD4_R$ donor cells recovered from the indicated SLOs of recipient mice are shown. **c,** Distribution of $CD4_N$, $CD4_M$ and $CD4_R$ cells among host and donor CD4 T cells recovered from the indicated SLOs of recipient mice. **d-f,** Percentages of $PD1^+$ (**d**), $KI67^+$ (**e**) and $TIGIT^+$ (**f**) cells among host and donor $CD4_M$ and $CD4_R$ cells are shown for the indicated SLOs of recipient mice. Quantifications are represented as Means ± SEM. The significance of differences between two series of results was assessed using Student's unpaired t test (**b** and **d-f**). Significant ($p < 0.05$) or almost significant ($0.05 < p < 0.10$) p-values are indicated. Source data are provided as a Source Data file.

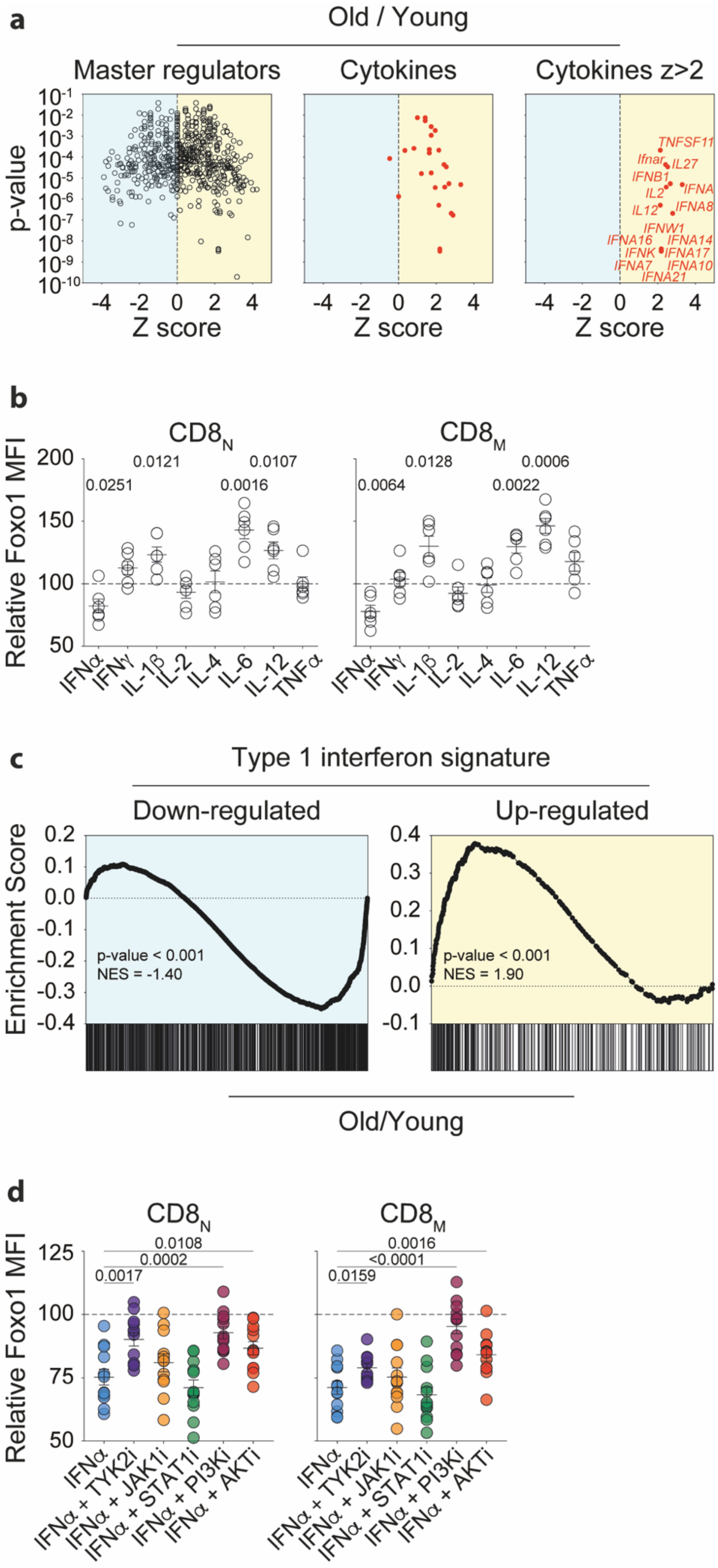
a
Old / Young
Master regulators
Cytokines
Cytokines z>2
p-value
Z score
TNFSF11
Ifnar
IL27
IFNB1
IL2
IFNA
IL12
IFNA8
IFNW1
IFNA16
IFNA14
IFNK
IFNA17
IFNA7
IFNA10
IFNA21
b
CD8N
CD8M
Relative Foxo1 MFI
0.0251
0.0121
0.0016
0.0107
0.0064
0.0128
0.0022
0.0006
IFNα
IFNγ
IL-1β
IL-2
IL-4
IL-6
IL-12
TNFα
c
Type 1 interferon signature
Down-regulated
Up-regulated
Enrichment Score
p-value < 0.001
NES = -1.40
p-value < 0.001
NES = 1.90
Old/Young
d
CD8N
CD8M
Relative Foxo1 MFI
0.0017
0.0002
0.0108
0.0159
<0.0001
0.0016
IFNα
IFNα + TYK2i
IFNα + JAK1i
IFNα + STAT1i
IFNα + PI3Ki
IFNα + AKTi

Figure S10

Fig. S10 Type 1 interferons modulate Foxo1 expression in T cells. a, p-value/Z score dot-plots representing all Master Regulators (left panel), the cytokines and cytokines-related regulators (middle panel) and among these ones, those with a Z score > 2 (right panel) determined by Ingenuity Pathway Analysis software as significantly ($p < 0.05$) involved in the transcriptomic signature of $CD4_N$ cells from old WT mice. **b**, Relative Foxo1 MFIs for $CD8_N$ and $CD8_M$ cells recovered after 4 days of culture with IL-7 alone or together with the indicated cytokines. Relative MFIs were calculated after barcoding by dividing the MFI of a given T-cell subset in the presence of one given cytokine by the MFI of the same T-cell subset cultured with IL-7 alone. **c,** GSEA analysis visualizing the expression of genes down- or up-regulated by type 1 interferons in T cells by $CD4_N$ cells from old versus young adult mice. **d,** Purified T cells from young WT mice were cultured for 4 days with IL-7 in the presence of IFNα4 and the indicated inhibitors. Relative MFIs were calculated after barcoding by dividing the MFI of a given T-cell subset in the presence of one given inhibitor and IFNα4 by the MFI of the same T-cell subset cultured with the same inhibitor alone. Statistics were calculated to compare Foxo1 expression level induced by IFNα4 alone with Foxo1 expression in the presence of the indicated inhibitors and IFNα4. Quantifications are represented as Means ± SEM. The significance of differences between two series of results was assessed using Student's unpaired t test (**a**, **b** and **d**). The p-values for the GSEA test statistics are calculated by permutation (**c**). Significant ($p < 0.05$) p-values are indicated. Source data are provided as a Source Data file.

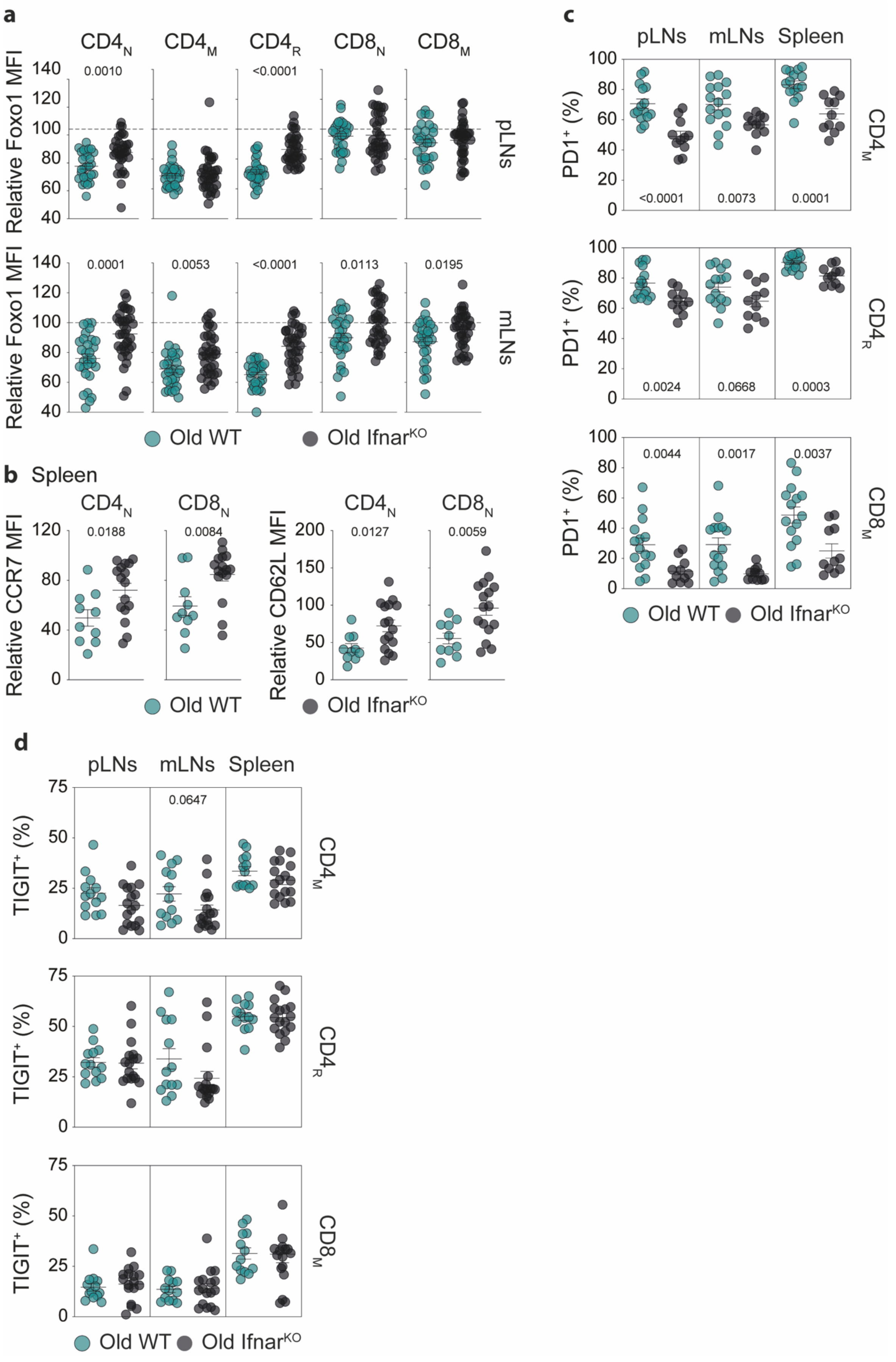

a
CD4N CD4M CD4R CD8N CD8M
Relative Foxo1 MFI
0.0010
<0.0001
pLNs
0.0001 0.0053 <0.0001 0.0113 0.0195
mLNs
Old WT
Old IfnarKO
b Spleen
CD4N CD8N
Relative CCR7 MFI
0.0188 0.0084
CD4N CD8N
Relative CD62L MFI
0.0127 0.0059
c
pLNs mLNs Spleen
PD1+ (%)
<0.0001 0.0073 0.0001
CD4M
0.0024 0.0668 0.0003
CD4R
0.0044 0.0017 0.0037
CD8M
d
pLNs mLNs Spleen
TIGIT+ (%)
0.0647
CD4M
CD4R
CD8M

Figure S11

Fig. S11 Comparison of the T-cell compartment of old WT and old Ifnar^{KO} mice. a, Cell suspensions from pLNs and mLNs of a 3-month-old WT mouse (Young adult), a 22-month-old WT mouse (Old) and a 22-month-old Ifnar^{KO} mouse (old) were first stained separately with anti- CD45 antibodies conjugated to different fluorochromes and then mixed before further staining. Relative Foxo1 MFIs were calculated by dividing the MFI of a given T-cell subset of old mice by the MFI of the same T-cell subset of the barcoded young adult WT mouse. **b**, Relative CCR7 and CD62L MFIs are shown for $CD4_N$ and $CD8_N$ cells recovered from the spleen of 22-month-old WT versus 22-month-old Ifnar^{KO} mice. **c,** Percentages of $PD1^+$ cells among $CD4_M$, $CD4_R$ and $CD8_M$ cells recovered from the indicated SLOs of 18-month-old WT versus 18-month-old Ifnar^{KO} mice. **d,** Percentages of $TIGIT^+$ cells among $CD4_M$, $CD4_R$ and $CD8_M$ cells recovered from the indicated SLOs of 22-month-old WT versus 22-month-old Ifnar^{KO} mice. Quantifications are represented as Means ± SEM. The significance of differences between two series of results was assessed using Student's unpaired t test (**a-d**). Significant ($p < 0.05$) or almost significant ($0.05 < p < 0.10$) p-values are indicated. Source data are provided as a Source Data file.
