# Supplementary material for: Type 1 interferons and Foxo1 down-regulation play a key role in age-related T-cell exhaustion in mice
Source: Nat Commun. 2024 Feb 26;15:1718. doi: 10.1038/s41467-024-45984-8 (PMC10897180; doi:10.1038/s41467-024-45984-8)
Supplement: Supplementary file 3 — Reporting Summary [file 41467_2024_45984_MOESM3_ESM.pdf]

nature portfolio

Corresponding author(s): Bruno LUCAS

Last updated by author(s): Jun 20, 2023

Reporting Summary

Nature Portfolio wishes to improve the reproducibility of the work that we publish. This form provides structure for consistency and transparency in reporting. For further information on Nature Portfolio policies, see our Editorial Policies and the Editorial Policy Checklist.

Statistics

For all statistical analyses, confirm that the following items are present in the figure legend, table legend, main text, or Methods section.

n/a	Confirmed	
☐	☒	The exact sample size (*n*) for each experimental group/condition, given as a discrete number and unit of measurement
☐	☒	A statement on whether measurements were taken from distinct samples or whether the same sample was measured repeatedly
☐	☒	The statistical test(s) used AND whether they are one- or two-sided<br>*Only common tests should be described solely by name; describe more complex techniques in the Methods section.*
☒	☐	A description of all covariates tested
☐	☒	A description of any assumptions or corrections, such as tests of normality and adjustment for multiple comparisons
☐	☒	A full description of the statistical parameters including central tendency (e.g. means) or other basic estimates (e.g. regression coefficient) AND variation (e.g. standard deviation) or associated estimates of uncertainty (e.g. confidence intervals)
☐	☒	For null hypothesis testing, the test statistic (e.g. *F*, *t*, *r*) with confidence intervals, effect sizes, degrees of freedom and *P* value noted<br>*Give P values as exact values whenever suitable.*
☒	☐	For Bayesian analysis, information on the choice of priors and Markov chain Monte Carlo settings
☒	☐	For hierarchical and complex designs, identification of the appropriate level for tests and full reporting of outcomes
☒	☐	Estimates of effect sizes (e.g. Cohen's *d*, Pearson's *r*), indicating how they were calculated

Our web collection on statistics for biologists contains articles on many of the points above.

Software and code

Policy information about availability of computer code

Data collection	Flow Cytometry: BD LSRFortessa (BD Biosciences)<br>Cell Sorting: BD FACSAria III (BD Biosciences)<br>Imaging Flow Cytometry: ImageStreamX MKII (Amnis)
Data analysis	- Flow Cytometry data were analyzed using DIVA 8.0.1 (BD Biosciences), Prism 8.2.1 (GraphPad) and Excel 15.29.1 (Microsoft)<br>- Images were analyzed using IDEAS 6.0 (Amnis), ImageJ 1.51m9, Prism 8.2.1 (GraphPad)<br>- Microarrays were analyzed analyzed with Expression Console software (Affymetrix) to obtain raw data (cel files) and metrics for Quality Controls. RMA normalization 89 was then performed using R. Entrez Gene CDF of Brain Array was used for normalization.<br>- For next generation sequencing, Fastq files were then aligned using STAR algorithm (version 2.7.6a), on the Ensembl Mus musculus GRCm38 reference, release 101. Reads were then count using RSEM (v1.3.1) and the statistical analyses on the read counts were performed with R (version 3.6.3) and the DESeq2 package (DESeq2_1.26.0) to determine the proportion of differentially expressed genes between two conditions. We used the standard DESeq2 normalization method (DESeq2's median of ratios with the DESeq function), with a pre-filter of reads and genes (reads uniquely mapped on the genome, or up to 10 different loci with a count adjustement, and genes with at least 10 reads in at least 3 different samples). Following the package recommendations, we used the Wald test with the contrast function and the Benjamini-Hochberg FDR control procedure to identify the differentially expressed genes. R scripts and parameters are availables on Github, https://github.com/GENOM-IC-Cochin/RNA-Seq_analysis/releases/tag/v1.202112.

For manuscripts utilizing custom algorithms or software that are central to the research but not yet described in published literature, software must be made available to editors and reviewers. We strongly encourage code deposition in a community repository (e.g. GitHub). See the Nature Portfolio guidelines for submitting code & software for further information.

Data

Policy information about availability of data

All manuscripts must include a data availability statement. This statement should provide the following information, where applicable:

- Accession codes, unique identifiers, or web links for publicly available datasets
- A description of any restrictions on data availability
- For clinical datasets or third party data, please ensure that the statement adheres to our policy

All data supporting the findings of this study will be available in a publicly accessible repository. The molecular signature datasets can be publicly accessed at https://www.ncbi.nlm.nih.gov/geo/ with accession codes GSE211827, .GSE211365 and GSE210191.

Human research participants

Policy information about studies involving human research participants and Sex and Gender in Research.

Reporting on sex and gender	*Use the terms sex (biological attribute) and gender (shaped by social and cultural circumstances) carefully in order to avoid confusing both terms. Indicate if findings apply to only one sex or gender; describe whether sex and gender were considered in study design whether sex and/or gender was determined based on self-reporting or assigned and methods used. Provide in the source data disaggregated sex and gender data where this information has been collected, and consent has been obtained for sharing of individual-level data; provide overall numbers in this Reporting Summary. Please state if this information has not been collected. Report sex- and gender-based analyses where performed, justify reasons for lack of sex- and gender-based analysis.*
Population characteristics	*Describe the covariate-relevant population characteristics of the human research participants (e.g. age, genotypic information, past and current diagnosis and treatment categories). If you filled out the behavioural & social sciences study design questions and have nothing to add here, write "See above."*
Recruitment	*Describe how participants were recruited. Outline any potential self-selection bias or other biases that may be present and how these are likely to impact results.*
Ethics oversight	*Identify the organization(s) that approved the study protocol.*

Note that full information on the approval of the study protocol must also be provided in the manuscript.

Field-specific reporting

Please select the one below that is the best fit for your research. If you are not sure, read the appropriate sections before making your selection.

[x] Life sciences [ ] Behavioural & social sciences [ ] Ecological, evolutionary & environmental sciences

For a reference copy of the document with all sections, see nature.com/documents/nr-reporting-summary-flat.pdf

Life sciences study design

All studies must disclose on these points even when the disclosure is negative.

Sample size	- In vivo experiments: no statistical method was used to determine sample size. The number of mice used was increased as the experiments progressed to improve statistical power.<br>- In vitro experiments: no statistical method was used to determine the sample size. A minimum of three independent experiments were performed. When this proved insufficient to clarify trends between groups, the number of experiments was increased to improve statistical power.
Data exclusions	No data were excluded from our analyses.
Replication	Experimental results were reliably replicated. For each type of experiment, the number of replications will be indicated in the figure legends.
Randomization	Mice were grouped according to age or genotype, and thus were not randomized.
Blinding	Blinding was not applied because mouse genotypes and their age were known before the experiments.

Reporting for specific materials, systems and methods

We require information from authors about some types of materials, experimental systems and methods used in many studies. Here, indicate whether each material, system or method listed is relevant to your study. If you are not sure if a list item applies to your research, read the appropriate section before selecting a response.

Materials & experimental systems

n/a	Involved in the study	
☐	☒	Antibodies
☒	☐	Eukaryotic cell lines
☒	☐	Palaeontology and archaeology
☐	☒	Animals and other organisms
☒	☐	Clinical data
☒	☐	Dual use research of concern

Methods

n/a	Involved in the study	
☒	☐	ChIP-seq
☐	☒	Flow cytometry
☒	☐	MRI-based neuroimaging

Antibodies

Antibodies used

Anti-mouse CCR7 BV605 Biolegend Cat# 120125; RRID:AB_2715777 (1/100)

Anti-mouse CD3e from hybridoma supernatant (145-2C11) our lab (0,1ug/mL)

Anti-mouse CD4 (GK1.5) BioXcell Cat#BE003; RRID:AB_1107642 (1/10)

Anti-mouse CD4 BV510 Biolegend Cat# 100559; RRID:AB_2562608 (1/200)

Anti-mouse CD4 Pacific Blue BD Biosciences Cat# 558107; RRID:AB_397030 (1/100)

Anti-mouse CD4 BV421 Biolegend Cat# 116023; RRID:AB_2800579 (1/200)

Anti-mouse CD4 PerCP-Cy5.5 BD Biosciences Cat# 550954; RRID:AB_393977 (1/100)

Anti-mouse CD8a APC-Vio770 Miltenyi Biotec Cat# 130-102-305; RRID:AB_2659897 (1/200)

Anti-mouse CD8a BV421 BioLegend Cat# 100753; RRID:AB_2562558 (1/1600)

Anti mouse-CD8a from hydridoma supernatant (53-6.7) our lab (0,02mg/mL)

Anti-mouse CD8b2 PE BD Biosciences Cat# 553041; RRID:AB_394577 (1/100)

Anti-mouse/human CD11b PE BD Biosciences Cat# 553311; RRID:AB_394775 (1/200)

Anti-mouse CD11b (MAC1) BioLegend Cat#101270; RRID:AB_2813919 (1/400)

Anti-mouse CD11c PE BD Biosciences Cat# 553802; RRID:AB_395061 (1/200)

Anti-mouse CD16/CD32 (2.4G2) BioXcell Cat# BE0307; RRID:AB_2736987 (5ug/mL)

Anti-mouse CD19 (1D3) BioXcell Cat# BE0150; RRID:AB_10949187 (1/400)

Anti-mouse CD19 PE BD Biosciences Cat# 557399; RRID:AB_396682 (1/200)

Anti-mouse CD25 PE BD Biosciences Cat# 553866; RRID:AB_395101 (1/200)

Anti-mouse CD39 PE eBioscience Cat# 12-0391-82; RRID:AB_1210740 (1/100)

Anti-mouse CD44 PE-Cy7 BD Biosciences Cat# 560569; RRID:AB_17274843 (1/200)

Anti-mouse/human CD44 BV785 BioLegend Cat# 103059; RRID:AB_2571953 (1/200)

Anti-mouse CD45 BUV395 BD Biosciences Cat# 564279; RRID:AB_2651134 (1/50)

Anti-mouse CD45 PE-Cy7 BD Biosciences Cat# 552848; RRID:AB_394489 (1/1600)

Anti-mouse CD45 BV711 BioLegend Cat# 103147; RRID:AB_2564383 (1/800)

Anti-mouse CD45 PerCP-Cy5.5 BD Biosciences Cat# 550994; RRID:AB_394003 (1/400)

Anti-mouse CD45.1 BV650 BioLegend Cat# 110735; RRID:AB_11124743 (1/50)

Anti-mouse CD45.2 Alexa Fluor 700 BioLegend Cat# 109822; RRID:AB_493731 (1/50)

Anti-mouse CD62L BV650 BD Biosciences Cat# 564108; RRID:AB_2738597 (1/100)

Anti-mouse Foxo1 Alexa Fluor 647 Cell Signaling Cat# 72874S; RRID:AB_2799829 (1/25)

Anti-mouse Foxo1 PE Cell Signaling Cat# 14262S; RRID:AB_2798437 (1/50)

Anti-mouse Foxo3 PE Cell Signaling Cat# 14592S; RRID:AB_2798529 (1/25)

Anti-mouse/Rat Foxp3 eBioscience Cat# 11-5773-82; RRID:AB_465243 (1/400)

Anti-mouse Histone H3 Alexa Fluor 647 Cell Signaling Cat# 12230S; RRID:AB_2797852 (1/25)

Anti-mouse/human Ki67 BV421 BioLegend Cat# 151208; RRID:AB_2629748 (1/1500)

Anti-mouse IL-17A V450 BD Biosciences Cat# 560522; RRID:AB_1727540 (1/50)

Anti-mouse NK1.1 PE BD Biosciences Cat# 553165; RRID:AB_394677 (1/100)

Anti-mouse PD1 PE eBioscience Cat# 12-9985-83; RRID:AB_466296 (1/100)

Anti-mouse phospho Foxo Cell Signaling Cat#9464; RRID:AB_329842 (1/100)

Anti-mouse phospho Akt Cell Signaling Cat# 4060; RRID:AB_2315049 (1/100)

Anti-mouse TCF-1 BV421 BD Biosciences Cat# 566692; RRID:AB_2869822 (1/100)

Anti-mouse TCRb PerCP-Cy5.5 eBioscience Cat# 45-5961-82; RRID:AB_925763 (1/200)

Anti-mouse TCRb Alexa Fluor 700 BioLegend Cat# 109224; RRID:AB_1027648 (1/50)

Anti-mouse TCRgd PE BD Biosciences Cat# 553178; RRID:AB_394689 (1/100)

Anti-mouse Ter-119 (TER-119) BioLegend Cat# 116255; RRID:AB_2832401 (1/500)

Anti-mouse TIGIT APC BioLegend Cat# 156106; RRID:AB_2750515 (1/50)

Anti-mouse TOX APC Miltenyi Biotec Cat# 130-118-335, RRID:AB_2751485 (1/200)

Validation	All antibodies were commerciale. Specificity and validation were provided by manufacturer's technical datasheets and confirmed in literature. No further validation was performed.

Animals and other research organisms

Policy information about studies involving animals; ARRIVE guidelines recommended for reporting animal research, and Sex and Gender in Research

Laboratory animals	3-month-old and 22-month-old mice were used for experiments unless otherwise indicated in the figure legends. C57BL/6 Foxp3-GFP CD45.1/2 or CD45.2 mice , C57BL/6 IfnarKO mice , C57BL/6 CD3KO and C57BL/6 Foxp3-GFP Foxo1TKO and Foxo1Ctrl CD45.1 or CD45.2 mice were maintained in our own animal facilities, under specific pathogen–free (SPF) conditions.
Wild animals	This study did not involve wild animals.
Reporting on sex	The old mice and their young controls were all female mice.
Field-collected samples	None
Ethics oversight	All procedures were approved by the ethics committee for animal experimentation n°34 and validated by the "Ministère de l'Enseignement Supérieur de la Recherche et de l'Innovation" with the number APAFIS #20630-2018033016303981v5.

Note that full information on the approval of the study protocol must also be provided in the manuscript.

Flow Cytometry

Plots

Confirm that:

- [x] The axis labels state the marker and fluorochrome used (e.g. CD4-FITC).
- [x] The axis scales are clearly visible. Include numbers along axes only for bottom left plot of group (a 'group' is an analysis of identical markers).
- [x] All plots are contour plots with outliers or pseudocolor plots.
- [x] A numerical value for number of cells or percentage (with statistics) is provided.

Methodology

Sample preparation

Cell suspensions. Peripheral lymph nodes (pooled cervical, axillary, brachial and inguinal lymph nodes; pLNs), mesenteric LNs (mLNs), spleens, and thymi were homogenized and passed through a nylon cell strainer in RPMI 1640 GlutaMAX supplemented with 10% FCS for adoptive transfer and cell culture (LNs only) or in 5% FCS, 0.1% NaN3 in phosphate saline buffer saline (PBS) for flow cytometry.

Fluorescence staining and flow cytometry.
Cell suspensions were collected and dispensed into 96-well round-bottom microtiter plates (6x106 cells/well). Surface staining was performed as described (Delpoux et al. 2014). Briefly, cells were incubated on ice, for 15 min/step, with Abs in 5% FCS, 0.1% NaN3 in PBS. Each cell staining reaction was preceded by a 15-min incubation with a purified anti-mouse CD16/32 Ab (FcgRII/III block; 2.4G2). The Foxp3 Staining Buffer Set was used for Foxp3, Foxo1, Foxo3, Ki-67, TOX and TCF-1 intracellular staining. This protocol was also used to determine intra + extracellular expression of CCR7. To assess pFoxo and pAKT levels ex vivo, spleen cells were immediately fixed in 4% PFA for 5 min at 37°C. Cells were then washed and permeabilized by adding ice-cold 100% methanol to a final concentration of 90% methanol and incubated for at least 30 min at -20°C. After extensive washing, cells were barcoded using anti-CD45 antibodies and stained for cell surface antigens. After an additional wash, intracellular antigens, including Phospho-Foxo1 (Thr24)/Foxo3 (Thr32) or Phospho-AKT (Ser473) (both from Cell Signaling), were stained overnight at 4°C. For determination of intracellular cytokine production, cells were stimulated with 0.5 ug/mL PMA, 0.5ug/mL ionomycin and 10ug/mL brefeldin A (all from Sigma-Aldrich) for 2h at 37°C. Cells were then stained for surface markers, fixed in 2% paraformaldehyde in PBS and permeabilized with 0.5% saponin, followed by labeling with specific cytokine Abs. Multi-color immunofluorescence was analyzed using a BD-Fortessa cytometer. Data acquisition and cell sorting were performed at the Cochin CYBIO facility. Throughout this study, as in our previous articles (Martin et al. 2013; Durand et al. 2018), regulatory CD4 T cells (CD4R) were defined as Foxp3+ CD4+ CD8b- TCRb+ cells, memory CD4 T cells (CD4M) as CD44hi Foxp3- CD4+ CD8b- TCRb+ cells and naive CD4 T cells (CD4N) as CD44-/low Foxp3- CD4 + CD8b- TCRb+ cells. CD44 expression was also used to discriminate between naive and memory CD8 T cells (CD8N and CD8M respectively).

Instrument

BD LSRFortessa (BD Biosciences), BD FACSAria III (BD Biosciences)

Software

DIVA 8.0.1 (BD Biosciences)

Cell population abundance

Sorted cells were >90% pure, as determined by FACS reanalysis.

Gating strategy

The gating strategy can be find in previous publications of the team (Durand, A. et al. Profiling the lymphoid-resident T cell pool reveals modulation by age and microbiota. Nat. Commun. 9, 68 (2018) ; Martin, B. et al. Highly self-reactive naive CD4 T cells are prone to differentiate into regulatory T cells. Nat. Commun. 4, 2209 (2013)).

- [x] Tick this box to confirm that a figure exemplifying the gating strategy is provided in the Supplementary Information.
