# Supplementary material for: Type 1 interferons and Foxo1 down-regulation play a key role in age-related T-cell exhaustion in mice
Source: Nat Commun. 2024 Feb 26;15:1718. doi: 10.1038/s41467-024-45984-8 (PMC10897180; doi:10.1038/s41467-024-45984-8)
Supplement: Supplementary file 4 — Description of additional supplementary files [file 41467_2024_45984_MOESM4_ESM.docx]

**Description of additional supplementary files**

**Supplementary Data 1:** Transcriptomic signature of CD4_N_ cells deficient for Foxo1 expression

**Supplementary Data 2:** Transcriptomic signature of LCMV specific CD4 T cells 30 days after chronic versus acute LCMV infection

**Supplementary Data 3:** Transcriptomic signature of LCMV specific CD4 T cells 8 days after chronic versus acute LCMV infection

**Supplementary Data 4:** Transcriptomic signature of activated human CD4_N_ cells as a function of age

**Supplementary Data 5:** Transcriptomic signature of CD4 T cells 2 hours after IFNα injection

**Supplementary Data 6:** ChIP-seq Foxo1 from naive CD4 T cells

**Supplementary Data 7:** Antibodies
